# Open-Pore Skeleton Prussian Blue as a Cathode Material to Achieve High-Performance Sodium Storage

**DOI:** 10.3390/ma18133174

**Published:** 2025-07-04

**Authors:** Wenxin Song, Yaxin Li, Jiahao Chen, Huihua Min, Xinyuan Wu, Xiaomin Liu, Hui Yang

**Affiliations:** 1 College of Materials Science and Engineering, Nanjing Tech University, Nanjing 211816, China; wenxinson@163.com (W.S.); liyaxin1101@163.com (Y.L.); chenjiahao1998@njtech.edu.cn (J.C.); wuxinyuan0315@163.com (X.W.); liuxm@njtech.edu.cn (X.L.); 2 Electron Microscope Lab, Nanjing Forestry University, Nanjing 210037, China; hhmin@njfu.edu.cn

**Keywords:** sodium-ion batteries, Prussian blue, open-pore skeletal structure, cathode

## Abstract

Prussian blue and its analogs (PBAs), considered potential cathode materials for sodium-ion batteries (SIBs), still confront multiple challenges. For example, many defect vacancies and high crystal water content are generated during the fast crystallization of PBAs, impairing the rate performance. The stress accumulation during Na^+^ insertion/extraction destabilizes the lattice framework and then damages the electrochemical performance. Herein, iron-based Prussian blue with an open-pore skeleton structure (PB-3) is prepared using a facile template method which employs PVP and sodium citrate to control the crystallization rate and adjust the particle morphology. The prepared materials exhibit excellent kinetic properties and are conducive to mitigate the volume changes during ion insertion/extraction processes. PB-3 electrode not only exhibits a superior rate performance (92 mAh g^−1^ reversible capacity at 2000 mA g^−1^), but also presents superior cycling performance (capacity retention remained at 90.2% after 600 cycles at a current density of 500 mA g^−1^). The highly reversible sodium ion insertion/extraction mechanism of PB-3 is investigated by ex situ XRD tests, which proves that the stabilized lattice structure can enhance the long cycling performance. In addition, the considerable capacitance contributes to the rate performance. This study provides valuable insights for the subsequent development of high-performance and stable cathodes for SIBs.

## 1. Introduction

Lithium-ion batteries (LIBs) have been used in a wide range of portable electronics and power grid applications, attributed to their exceptional energy density and prolonged cycling stability [1]. The demand for Lithium has increased dramatically since the annual production of Li-ion batteries exceeded 1 TWh in 2023. Moreover, the uneven and limited distribution of Lithium resources is exacerbating people’s concerns, and has therefore brought an urgent need to develop an alternative secondary type of battery using an earth-abundant element. In this case, sodium-ion batteries (SIBs) have gradually emerged as a highly promising substitute for LIBs, benefiting from the earth’s abundant sodium reserves and nearly identical electrochemical reaction mechanisms [2]. The development of cathode materials with excellent electrochemical properties and cost-effective performance has become an urgent task to promote the development of SIB technology.

The electrochemical performance of SIB is limited by the ionic radius of Na^+^ and the electronic/ionic conductivity of the electrode material [3,4]. Consequently, the development of cathode materials with excellent electrochemical properties has become a key challenge. To date, extensive investigations have focused on high-performance cathode systems, especially polyanionic compounds [5,6], layered metal oxides [7,8], and Prussian blue analogs (PBAs). PBAs have an open framework structure. They have a large number of interstitial sites for Na^+^ diffusion, low synthesis cost, and high theoretical specific capacity, and are thus considered the most promising SIB cathode materials [9,10,11]. PBAs can be expressed as Na_x_M[Fe(CN)_6_]_1−y_☐_y_·nH_2_O (0 < x ≤ 2, 0 < y < 1), where M represents transition metal elements such as Fe, Mn, Co, Ni, Cu, and Zn, and ☐ represents [Fe(CN)_6_]^4−^ vacancies. Despite the many advantages of PBAs, there are still some constraints for developing superior cathode materials. The excessively fast reaction rate during the precipitation of PBAs leads to the presence of vacancy defects and crystallization of water in the lattice, resulting in unsatisfactory battery performance [12,13,14,15]. Due to the presence of [Fe(CN)_6_]^4−^ vacancies, the lattice structure of the material is prone to collapse during the Na^+^ insertion/extraction process, which leads to a decrease in the cycling performance of the electrode. The crystallization of water affects the insertion/extraction of sodium ions and blocks the movement of electrons, leading to poor rate performance and poor electronic conductivity [16,17,18]. What is more, the shrinkage/expansion of the crystal structure during Na^+^ insertion/extraction cause a heavy blow to the structural stability of PBAs [19].

Researchers have made significant efforts to solve the above issues. In order to obtain an intact crystal structure, chelating agents such as sodium citrate, disodium ethylenediaminetetraacetate, and sodium carboxymethylcellulose were added during the preparation process, which could slow down the reaction rate to reduce the vacancy defects [20,21,22]. In addition, the improvement of electronic conductivity and rate capacity can be achieved by increasing the contact interface between the active material and the electrolyte. For example, the PBAs with particular morphologies such as macroporous/mesoporous, dual-textured structure, and stepwise hollow structure shorten ion diffusion paths to some extent and possess a large void space to solve the problems caused by volume changes [23,24,25]. However, these unique structures are basically formed by acid etching, which may bring more defects, leading to the lower crystallinity of the material and the generation of the toxic substance HCN, which is unfavorable for practical applications.

Herein, a safe and facile co-precipitation method to synthesize iron-based Prussian blue with open-pore skeleton architecture cathode materials for SIBs is reported. By incorporating surfactant (PVP) and chelating agent (sodium citrate) with optimized ratio regulation, the as-synthesized cathode materials featuring high sodium content and low crystalline water exhibit outstanding cycling stability and rate capability. Meanwhile, this structure not only provides a larger contact area with electrolytes for electron transport and ion diffusion but also reserves buffer space for volume expansion [26]. A series of electrochemical characterizations confirmed that the as-prepared sample (PB-3) exhibited remarkable long-term cycling performance and superior rate performance. Specifically, the capacity retention remained at 90.2% after 600 cycles at a current density of 500 mA g^−1^, and maintained a specific capacity of 92 mAh g^−1^ even at 2000 mA g^−1^. Moreover, the storage mechanism and crystal structure evolution of Na^+^ during charge/discharge were analyzed by in situ XRD.

## 2. Experimental Methods

### 2.1. Materials Synthesis

The iron hexacyanoferrate (FeHCF) was synthesized via a facile co-precipitation method. Initially, 3 g polyvinylpyrrolidone (PVP, K30, with an approximate molecular weight of 40,000) and 10 g sodium citrate (C_6_H_5_Na_3_O_7_) were dissolved in 50 mL deionized water, forming Solution I. After 30 min of magnetic stirring, 3 mmol FeSO_4_·7H_2_O was added to Solution A, followed by an additional 30 min of stirring. Concurrently, 1 mmol Na_4_Fe(CN)_6_ was dissolved in 50 mL of deionized water to form a clear yellow Solution II, which was stirred for 30 min. Solution II was added dropwise to Solution Ⅰ using a peristaltic pump, followed by vigorous magnetic stirring for 12 h. After aging at room temperature (25 °C) for 24 h, the precipitate was collected by sequential washing with deionized water and ethanol (three times each), and finally dried in a vacuum oven at 100 °C for 12 h. The obtained sample was marked as PB-3. For comparison, the amount of C_6_H_5_Na_3_O_7_ addition was varied by adding 4 g, 7 g, and 13 g, and the samples obtained were marked as PB-1, PB-2, and PB-4, respectively. PVP and FeSO_4_·7H_2_O are from Sinopharm Group Chemical Reagent Co., Ltd., Shanghai, China. Deionized water is obtained from McLean Biochemicals Co., Ltd., Shanghai, China. C_6_H_5_Na_3_O_7_ is from Shanghai Aladdin Biochemical Technology Co., Ltd., Shanghai, China. Na_4_Fe(CN)_6_ is from Shanghai Merair Chemical Technology Co., Ltd., Shanghai, China.

### 2.2. Material Characterization

The FeHCF crystal structure of the materials was analyzed using X-ray diffraction (SmartLab-TM-3KW X-ray diffractometer, Rigaku, Japan). The elemental molar ratios in FeHCF were determined using direct-reading inductively coupled plasma optical emission spectrometry (ICP-OES, Optima 4300DV, PerkinElmer, USA). In addition, the inorganic coordination ions and organic functional groups in the framework were described via Fourier transform infrared (FT-IR) spectroscopy (Nicolet iS20, Thermo Scientific, USA). X-ray photoelectron spectra (XPS, Ne xsa, ThermoFisher, USA) was used to perform the element valences. Thermo gravimetric (TG, Netzsch STA449F5, Netzsch, Germany) were performed to investigate the water content in the samples’ compounds. The different morphologies were investigated by scanning electron microscopy (8100, Regulus, Japan) integrated with an energy dispersive spectrometer (EDS), enabling elemental mapping analysis for compositional characterization. The Nitrogen adsorption–desorption isotherms (ASAP 2460, Micromeritics, USA) was performed with a Specific Surface Area.

### 2.3. Electrochemical Measurements

The Cathode electrodes (mass loading of 2.27 mg cm^−2^) were prepared by mixing the FeHCF (70%), Super-P conductive (20%), and polymer binder (polyvinylidene fluoride, PVDF) (10%). The well-dispersed slurry was cast onto copper foil and vacuum-dried at 100 °C for 12 h. The pole piece was cut into 13 mm diameter circular discs. The prepared electrodes were assembled into a coin cell in an Ar-filled glove box (O_2_ and H_2_O concentrations less than 0.01 ppm). Galvanostatic electrochemical tests were conducted on a Neware workstation within 2.0–4.2 V (vs. Na^+^/Na). Electrochemical impedance spectroscopy (EIS) was performed using a CHI660e workstation at open circuit voltage (OCV) from 0.01 Hz to 0.1 MHz. Cyclic voltammetry (CV) was performed over a voltage range of 2 to 4.2 V using an electrochemical workstation (CHI660E, Beijing Huayan High-Tech, China).

## 3. Results and Discussion

**Figure 1:**
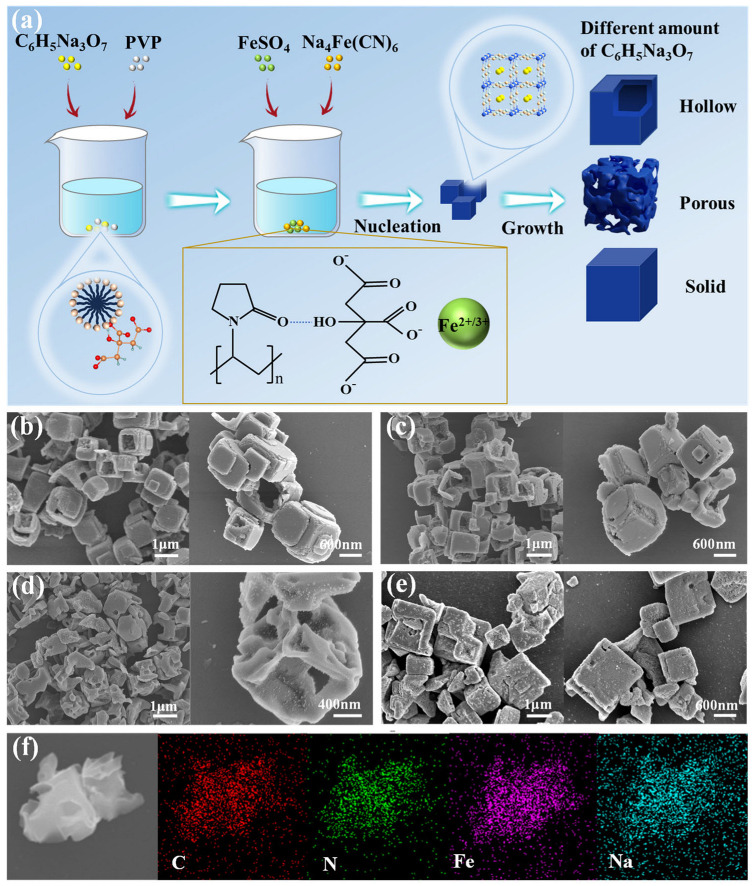
(**a**) Schematic illustrations of the synthesis process and architecture of FeHCF; SEM of (**b**) PB-1; (**c**) PB-2; (**d**) PB-3 and (**e**) PB-4; (**f**) EDS element mappings of PB-3.

As shown in Figure 1a, the synthesis process of different forms of FeHCFs is schematically illustrated. In the solution, the PVP formed micelles were bound to citrates due to the electrostatic interaction, which then could work as templates to absorb Fe^2+^. Meanwhile, Fe^2+^ could chelate with citrates to reduce the nucleated rate of FeHCF through the competitive reaction with ferrous cyanides [27]. As shown in Figure 1b–e, different ratios of PVP to sodium citrate give different forms of FeHCF. When the ratio of PVP to sodium citrate is 3:4 (Figure 1b) or 3:7 (Figure 1c), the resulting materials show hollow cubic morphologies with particle sizes between 700 and 900 nm. In addition, the shells of some cubic particles are disappeared with their cavities exposed. When the ratio of PVP to sodium citrate is 3:10, the PB-3 particles show a dispersed cubic morphology with an open-pore skeletal structure (Figure 1d). The formation of this structure might be due to the addition of different amounts of sodium citrate, which changes the morphology of the micelles [28,29].

This special morphology might have several advantages in the application of sodium-ion batteries. The large specific surface area of such architecture affords abundant active sites, facilitating the participation of Fe^2+^/Fe^3+^ in redox reactions. Meanwhile, the shortened Na^+^ diffusion pathway significantly enhances electrochemical kinetics. Additionally, this structure effectively mitigates the volumetric fluctuations, thus improving the stability of the FeHCF frame structure [30,31,32]. Interestingly, when the corresponding ratio of PVP to sodium citrate is 3:13, the morphology shows a solid cubic structure as shown in Figure 1e. This may be due to a large amount of sodium citrate addition making the PVP salt-out, resulting in the loss of its template function [29,33]. The corresponding EDS mapping images of PB-3 (Figure 1f) demonstrate that four elements, C, N, Fe, and Na are uniformly distributed in the particles, further indicating the high purity and structural integrity of the crystal.

**Figure 2:**
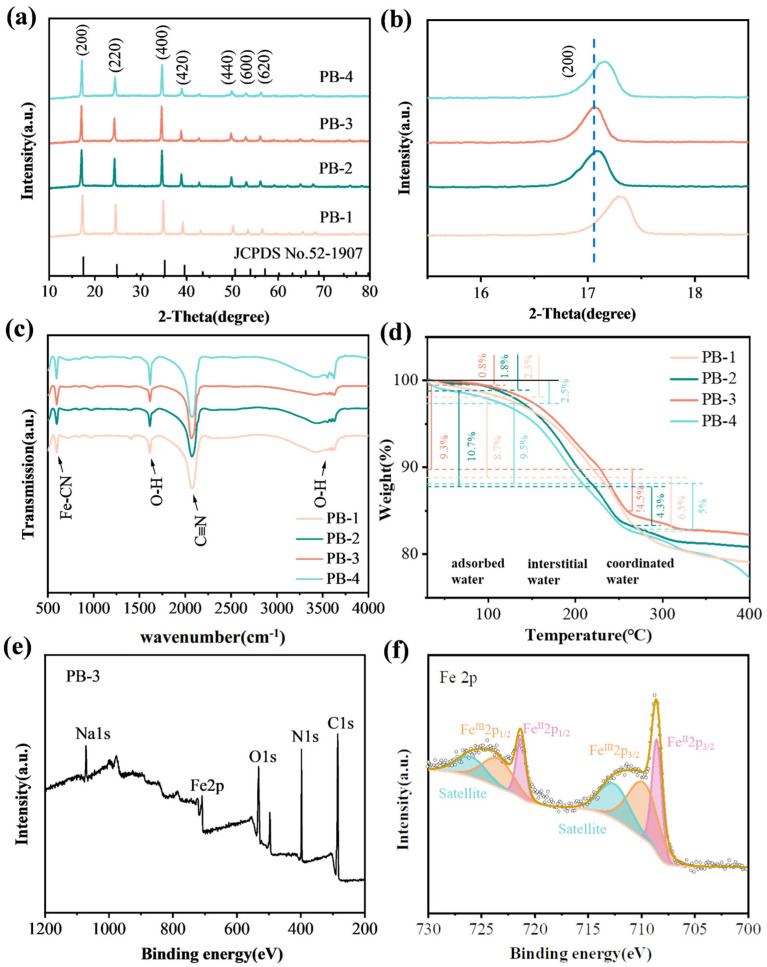
(**a**) XRD plots; (**b**) enlarged patterns; (**c**) FT-IR spectra and (**d**) TG curves of different materials; (**e**) XPS surveys of PB-3; (**f**) Fe 2p spectrum of PB-3.

Figure 2a shows the XRD plots of PB-1, PB-2, PB-3, and PB-4. All peaks at 2θ = 17.06°, 24.26°, 34.54°, 38.82°, 49.78°, 52.94°, and 56.1°, corresponding to the (200), (220), (400), (420), (440), (440), (600), and (620) lattice planes, show good agreement with the standard pattern (JCPDS No. 52-1907). This confirms that the space group is a face-centered cubic (FCC) structure of Fm-3m. Figure 2b shows the magnified view of the diffraction peaks of crystal planes (200), in which PB-3 presents the largest left shift in the diffraction peaks, illustrating that it has the largest inter-planar spacing [34]; with α = β = γ = 90° and the typical cubic structure, PB-3 has the largest refined lattice parameter of a = b = c = 10.3576 Å (10.2940 Å, 10.3375 Å, and 10.3225 Å for PB-1, PB-2, and PB-4, respectively) (Table S2). These phenomena are ascribed to the high Na^+^ content in the pristine lattice of PB-3, which effectively expands the lattice framework. Larger crystal plane spacing is more favorable for sodiation/disodiation process.

Figure 2c reveals that the four samples exhibit prominent absorption peaks primarily at 594.1 cm^−1^, 1618.3 cm^−1^, 2073.7 cm^−1^, and 3593.3 cm^−1^. In the infrared spectrum, the bands at 594.1 cm^−1^ and 2073.7 cm^−1^ can be attributed to the in-plane bending vibration and symmetric stretching vibration of the Fe-C≡N^−^ moiety, respectively. The absorption bands at 1618.3 cm^−1^ and 3593.3 cm-^1^ correspond to the bending of the O-H bond and the stretching of the H-O bond in the H-O-H plane, respectively, which are related to the crystallization of water. In particular, PB-3 reveals the smallest peak at 3593.3 cm^−1^, which hints that PB-3 has less interstitial water and coordinated water in the crystal framework of PBAs.

The chemical composition of four samples (PB-1, PB-2, PB-3, and PB-4) were analyzed via ICP-MS and TGA. A detailed elemental analysis of the PBA materials is presented in Table S1. To further quantify the water content in the as-prepared materials, TGA was performed by heating from 25 °C to 400 °C at rate of 10 °C min^-1^ under N_2_ atmosphere (Figure 2d). The weight loss is divided into three stages: adsorbed water on the surface of the particles, interstitial water, and coordinated water in the vacancies [35,36]. The mass loss below 100 °C is due to the loss of adsorbed water, while the interstitial and allotropic water is mainly lost in the range of 100–260 °C. The results show that the weight loss of four samples in that temperature region is 15.2%, 15%, 13.8%, and 14.5%, respectively, which indicates that PB-3 possesses perfect crystallinity with less crystallization of water and fewer vacancies in its crystal structure. PBAs with water content below 15% generally exhibit superior rate capability and cycle durability. Based on the obtained atomic occupancy (Table S2) and TGA results (Figure 2d), the chemical formula of PB-3 can be expressed as Na_1_._64_Fe[Fe(CN)_6_]_0_._98_ 2.67H_2_O.

As shown in Figure 2e, the survey spectra confirm the presence of C, O, Na, Fe, and N in the material. In the Fe 2p spectrum (Figure 2f), the Fe^II^2p3/2 and Fe^II^2p1/2 are observed at 708.58 eV and 721.38 eV, respectively, while the peaks at 710.08 eV and 723.58 eV can be assigned to Fe^III^2p3/2 and Fe^III^2p1/2. The Fe^3+^ species likely originate from the inevitable oxidation of Fe^2+^ during synthesis and washing due to air exposure. Based on the morphological characteristics, nitrogen adsorption–desorption measurements were conducted on PB-2 and PB-3 to characterize their surface states.

The N_2_ adsorption–desorption curves and pore size distribution curves of PB-2 and PB-3 are shown in Figure S2. The pore size distribution of PB-2 and PB-3 ranges from 2 to 55 nm, with PB-3 exhibiting a larger average pore size. Specifically, PB-3 has a BET specific surface area of 12.766 m^2^ g^−1^, which is significantly higher than PB-2′s 8.565 m^2^ g^−1^. This larger specific surface area facilitates thorough contact between the electrolyte and active materials, enhancing the sufficiency of interfacial reactions. Consequently, it promotes the insertion/extraction of Na^+^ and electron transport, thereby improving the battery’s kinetic performance.

The electrochemical performances of four FeHCF samples were evaluated. CV scans were performed over a voltage range of 2 to 4.2 V at a scan rate of 0.1 mV s^−1^. The four redox peaks corresponded to the sodiation/de-sodiation of Na^+^ at different positions/locations within the crystal structure [37]. The two pairs of redox peaks at lower potentials, for example, 3.11/2.85 V and 3.43/3.31 V for PB-3, are ascribed to the redoxes of Fe^HS^ coordinated to N, corresponding to the extraction/insertion of Na^+^ at 8c and 24d sites, respectively. The hollow or porous structure of the PB-1, PB-2, and PB-3 electrodes facilitates the observation of redox peaks at higher voltages (above about 4.0 V), which are associated with the recurrent formation and decomposition of the solid electrolyte interphase (SEI) layer. In contrast, the PB-4 electrode exhibits fewer side reactions under the same high-voltage conditions, indicating a more stable interfacial behavior. As cycling proceeds within 2.0–4.2 V under galvanostatic charge/discharge, the peak currents gradually decline and stabilize, suggesting the progressive formation and eventual stabilization of the SEI layer [38].

**Figure 3:**
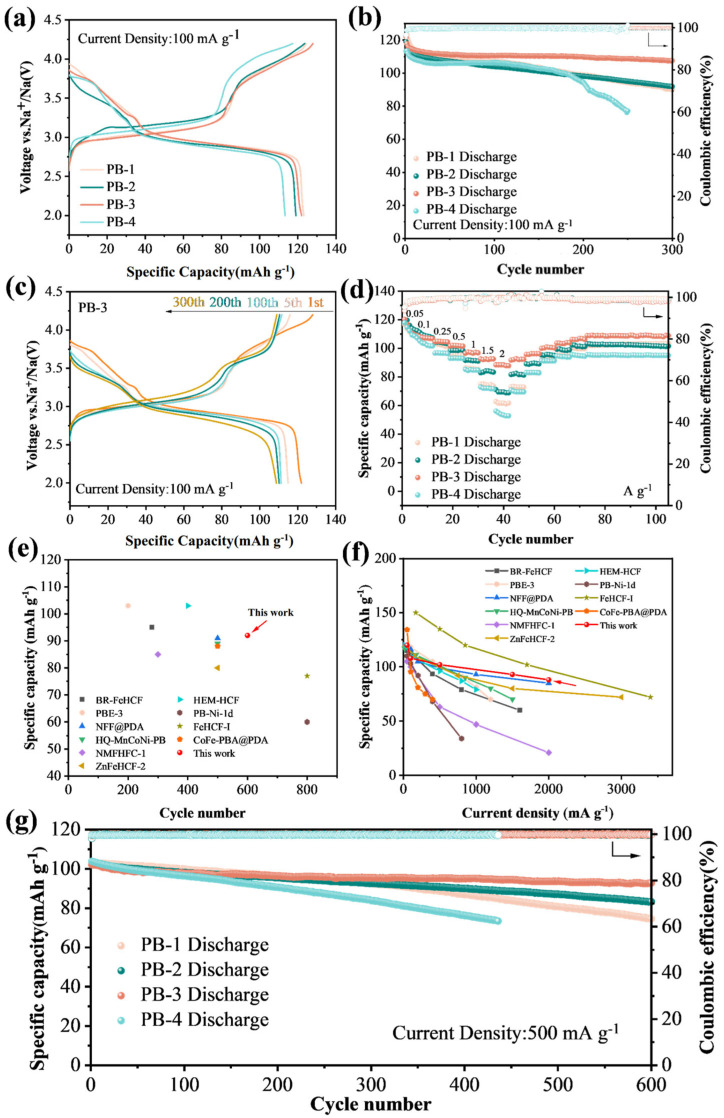
Electrochemical performance of four samples. (**a**) Cycle charge/discharge curve; (**b**) constant current discharge test at 100 mA g^−1^; (**c**) charge/discharge curves of the PB-3 electrode at 100 mA g^−1^; (**d**) rate performances of different four samples; (**e**) cycling performance and (**f**) rate performance of PB-3 electrode compared to previously reported Prussian blue cathodes; (**g**) long cycle performance at 500 mA g^−1^.

When being cycled at 100 mA g^−1^, PB-1, PB-2, PB-3, and PB-4 delivered the first discharge specific capacity of 123.07, 118.99, 121.98, and 113.38 mAh g^−1^ (Figure 3a). The long plateau observed in the low voltage range around 3 V (which is consistent with the large redox peaks detected in the CV curves of all four samples). Figure 3b illustrates the cycling performance of the four samples at 100 mA g^−1^ for all four samples. Among them, PB-3 demonstrates the best cycling stability, retaining 108.5 mAh g^−1^ with 89.0% capacity retention after 300 cycles. In contrast, PB-1, PB-2, and PB-4 maintain only 73.1%, 77.2%, and 68.1% of their initial capacities, respectively, over the same period. The superior durability of PB-3 is likely attributed to its stable crystal structure and reduced crystal water content. As shown in Figure 3c, the capacity decay of the electrode mainly occurs in the first 50 cycles or so, which may be attributed to the high specific surface area, leading to more surface side reactions and further formation of a passivation layer on the surface. The stability during the subsequent cycles is due to the fact that this passivation layer reduces the erosion of the material surface by the electrolyte and ensures the stability of the structure [39].

The dQ/dV curves were used to further analyze the reasons for the different cycling performance of the four samples, as shown in Figure S4. The dQ/dV curves of PB-3 have good overlap in the first two hundred cycles compared to other samples. Moreover, the redox peaks of the other samples decayed faster at a lower voltage during cycling, which may be due to the fact that some of the Fe^HS^ connected to N dissolved in the electrolyte, resulting in a reduction in the capacity provided at this voltage, whereas PB-3 has a more stable crystal lattice structure and Fe^HS^ is essentially undissolved.

In addition, PB-3 exhibits an excellent rate performance at 0.05 to 2 A g^−1^. At 0.05 A g^−1^, the PB-3 exhibits multiple redox reaction plateaus with a capacity of 120.3 mA h g^−1^ (Figure S5). Upon increasing the current density to 0.1, 0.25, 0.5, 1.0, 1.5, and 2.0 A g^−1^, the electrode delivers the capacities of 108.2, 104.8, 102.1, 97.4, 92.6, and 88.4 mAh g^−1^, respectively. Moreover, the capacity of 109 mAh g^−1^ remains after the current density is returned back to 50 mA g^−1^ stepwise, and it can still hold a stable state in the subsequent cycles, indicating that the crystal can still maintain a stable structure under the rapid removal and insertion of sodium ions (Figure 3d). On the other hand, the rest three samples display poor rate capacity, as seen in Figure 3d and Figure S5. Compared with PB-3, more capacity loss was observed at the voltage plateau, especially the redox peaks at high potentials almost disappearing, indicating a slower redox reaction rate at high current density. Figure 3g shows the long-cycle performance of four different samples at 500 mA g^−1^, in which PB-3 exhibits the best performance. PB-3 delivers an initial capacity of 102 mAh g^−1^, and retains 92 mAh g^−1^ after 600 cycles, corresponding to a capacity retention of 90.2% and a per-cycle capacity loss of only 0.016%. Compared with the previously reported PBA cathodes for sodium-ion batteries (Figure 3e,f), PB-3 also has certain advantages in terms of the electrochemical performance [30,32,38,40,41,42,43,44,45,46]. Particularly in terms of long-cycle stability, we compared previously reported cathode materials for PBAs (Table S4). The PB-3 electrode exhibits very high capacity retention.

The inter-surface resistance and electron transport capacity were investigated by utilizing electrochemical impedance spectroscopy (EIS) (Figure S6 and Table S2). The EIS spectra of the four samples display typical Nyquist plot features, comprising a semicircular arc in the high-frequency region and a linear segment with a 45° slope in the low-frequency domain. The high-frequency semicircle, attributed to the charge transfer resistance (R_ct_) at the electrode/electrolyte interface, reflects the kinetic barrier for ion-electron transfer, while the low-frequency inclined line—arising from the Warburg impedance (Z_w_)—signifies the diffusion-controlled ion transport through the electrode’s porous structure. This behavior is characteristic of mixed capacitive-diffusive charge storage mechanisms in electrochemical systems. The small intercept observed at the high-frequency end represents the cell’s bulk resistance (R_s_), which reflects the combined ionic/electronic conductivity contributions from the electrolytes, electrodes, and separator. This indicates that the R_ct_ of PB-3 is 92.96 Ω, which is lower than that of the other samples. Moreover, the slope of PB-3 at a low frequency is also higher, indicating it has a better kinetics for ionic conductivity. The above results suggest that PB-3 may have fewer vacancy defects, which facilitates the transport of electrons and ions. At the same time, the special pore structure reduces the ion diffusion distance and accelerates the ion transport process.

**Figure 4:**
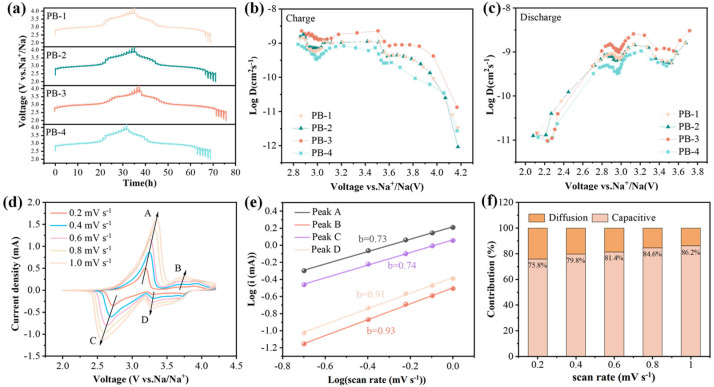
(**a**) GITT profiles of four samples; (**b**) Na^+^ diffusion coefficient during charging process; (**c**) Na^+^ diffusion coefficient during discharge process; (**d**) cyclic voltammetry curves of PB-3 (A, B, C, and D represent the oxidation and reduction peaks in the CV curve of PB-3, respectively); (**e**) curve after linear fitting; (**f**) contribution ratios from the diffusion-controlled and capacitive processes of PB-3 at different scan rates.

The galvanostatic intermittent titration technique (GITT) was employed to measure the solid-state diffusion coefficient of Na^+^ (D_Na+_) in PB-1, PB-2, PB-3, and PB-4 electrodes. During the cycling, a constant current pulse of 25 mA g^−1^ was applied for 10 min (τ), followed by a 60 min rest period to allow the cell to relax and stabilize at the open-circuit voltage (Es) [47]. The sodium-ion diffusion coefficient was calculated based on Fick’s second law, using the following equation:

(1) $$D_{{Na}^{+}}=\frac{4}{\pi \tau }{\left(\frac{n_{B}V_{m}}{S}\right)}^{2}{\left(\frac{\Delta E_{s}}{\Delta E_{\tau }}\right)}^{2}$$

where τ, n_B_, V_m_, and S represent the pulse time, the molar mass of the active material, the molar volume and the electrode interfacial area, respectively. $\Delta E_{s}$ is the voltage drop between the initial and steady states and $\Delta E_{\tau }$ is the change in cell voltage during a constant pulse time. Based on the equation, diffusion coefficients of sodium ions with voltage changes during charge and discharge were calculated, as shown in Figure 4b,c. The sodium ion diffusion coefficients of the four samples show a similar trend in relation to the voltage, and there are two characteristic V-shaped profiles in both charge and discharge stages, respectively, indicating that redox reactions occur at this voltage. Compared with other samples, PB-3 exhibits a higher D_Na_^+^. From the figure, it can be seen that PB-3 has the highest sodium ion diffusion coefficient values compared to those of other samples, with the highest value of 2.28 × 10^−9^ cm^2^ s^−1^ and 3.05 × 10^−9^ cm ^2^ s^−1^ in the charge/discharge process, respectively. Consequently, PB-3 exhibits a better Na^+^ transport kinetic in the lattice structure, which decreases the electrochemical polarization during charge and discharge process, leading to an outstanding rate performance of PB-3.

The CV curves of PB-3 at different sweep rates were tested to understand the underlying storage mechanism responsible for the superior electrochemical performance of PB-3. Figure 4d shows the cyclic voltammetry (CV) curve for PB-3 at 2.0–4.2 V (at sweep rates of 0.2, 0.4, 0.6, 0.8, and 1.0 mV s^−1^). Redox kinetics stands as a pivotal facet in unraveling the electrochemical behavior of electrode materials. To dissect this intricate process, we turn to the power-law relationship governing the interplay between peak current (i) and scan rate (v). This fundamental connection, articulated by the subsequent equations, serves as a cornerstone for quantitative analysis:

(2) $$i=av^{b}$$

(3) $$\log\left(i\right)=b\;log\left(v\right)+log(a)$$

where i denotes the experimentally measured current response reflecting the charge-transfer dynamics at the electrode/electrolyte interface, and a and b represent adjustable fitting parameters that encapsulate the intrinsic electrochemical characteristics. After the b value is calculated from the tangent of log (v) and log (i), it is used to determine whether there is pseudocapacitive behavior of the electrode material during charge/discharge process. When the b value approaches or is above 1, it indicates that the sodium storage mechanism is controlled by capacitance; on the contrary, the b value is close to 0.5, which indicates a solid-state diffusion process. To quantitatively dissect the electrochemical response at a specific scan rate, the respective contributions of pseudocapacitive behavior ($k_{1}v$) and diffusion-controlled processes ($k_{2}v^{1/2}$) can be deconvoluted using the following mathematical formulation. This approach enables precise differentiation between surface-dominated charge storage and bulk diffusion-limited dynamics [48]:

(4) $$i=k_{1}v+k_{2}v^{1/2}$$

As shown in Figure 4f, the capacitive contribution of PB-3 increases gradually with the increment of scan rate, which are 75.8%, 79.8%, 81.4%, 84.6%, and 86.2% at 0.2 to 1 mV s^−1^, respectively. The ion battery with capacitive behavior has extremely fast electrochemical reaction kinetics, which can effectively improve its cycling stability and rate performance; thus, capacitive behavior is also responsible for the excellent electrochemical performance of PB-3 [49].

**Figure 5:**
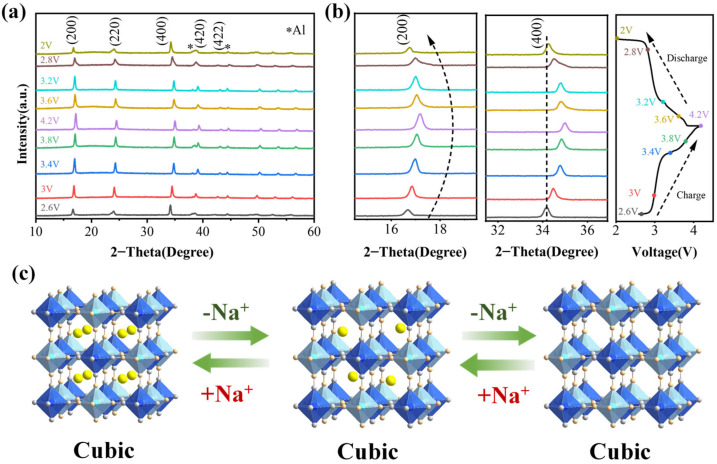
Investigation of phase transitions of PB-3 porous skeleton with cubic structure during cycling (* represents the diffraction peak of the Al foil in the electrode). (**a**) In situ XRD results of PB-3 under different charging and discharging states; (**b**) zoomed-in image showing the shifts in the (200) and (400) diffraction peaks (left) and the corresponding charging and discharging voltage profiles (right); (**c**) schematic representation of the structural phase transition during charging and discharging.

To reveal the sodium storage mechanism of PB-3 and to analyze the intrinsic reasons for the excellent electrochemical performance of this material at the crystal structure level, an ex situ XRD test was carried out within 2.0–4.2V (Figure 5a). As the charging process progresses, the diffraction peaks corresponding to the (200), (220), (400), (420), and (422) planes exhibit a gradual shift toward higher angles. Concurrently, the peaks at 16.8° and 24.5° show increased intensity. These observations indicate lattice contraction, likely caused by internal structural strain resulting from the extraction of sodium ions from the crystal framework. During the subsequent discharge progress, the diffraction peaks gradually shift towards lower 2θ values, and the lattice parameter gradually increases. It is noteworthy that the diffraction peaks of (200) and (400) return to the position of the initial charge at the end of the discharge, as shown in the magnified image (Figure 5b). At the same time, the diffraction peaks do not split significantly (except for 2.6 V and 2 V) and always maintain the cubic phase. This behavior can be attributed to the progressive reduction in lattice symmetry within the original cubic structure as Na^+^ ions accumulate in the lattice. The electrochemical potential variation induces a crystallographic phase transition from the cubic to rhombohedral structure, evidenced by the splitting of the (220) X-ray diffraction peak. A quantitative analysis reveals that charging to 2.6 V causes the (220) peak to decompose into two well-resolved peaks at 2θ = 18.2° and 18.8°, corresponding to the (111) and (222) reflections of the rhombohedral phase, respectively. Conversely, discharging to 2.0 V results in the recombination of these peaks, confirming the reversibility of the phase transition. This splitting phenomenon arises from the lattice parameter anisotropy during the cubic-to-rhombohedral transformation. The well-maintained lattice symmetry in the initial cubic framework (Figure 5c) suggests that the insertion/extraction of Na+ into the PB-3 lattice is highly reversible, which is favorable for the stability of the crystal structure during charging and discharging [43,50].

Figure S7 shows the comparison of SEM and XRD before and after 400 cycles of PB-3. The post-cycling SEM shows a smooth and flat surface of the pole piece compared with the pre-cycling one, with no large cracks. Furthermore, the particle morphology and particle size are basically consistent with the pre-cycling one. Similarly, PB-3 remained in the cubic phase with a complete crystal structure after 400 cycles. The XRD of PB-3 was subjected to Rietveld refinement (Figure S8) to obtain information such as lattice constants and atomic occupation (Table S5). The post-cycling XRD analysis showed that the (200) peak of PB-3 retained the lattice parameter of 10.3512 Å, which was only 0.06% lower than the original value (10.3576 Å), and the [Fe(CN)_6_]^4−^ vacancy was reduced from 0.2 to 0.19, confirming the minimal lattice distortion.

## 4. Conclusions

In summary, iron-based Prussian blue with an open-pore skeleton structure was prepared by the surfactant PVP together with appropriate amount of chelating agent sodium citrate. On one hand, the reaction rate during synthesis was reduced and a more perfect crystal structure was obtained. On the other hand, this remarkable structure increases the contact area between the electrode material and the electrolyte and at the same time alleviates the strain accumulation due to lattice volume changes during Na^+^ insertion/extraction. The as-synthesized PB-3 maintains a reversible cubic phase with small volume variation, and significantly inhibits the metal dissolution. As a consequence, the PB-3 has an outstanding rate performance, still providing an 88.4 mAh g^−1^ specific capacity at 2000 mA g^−1^ and a capacity retention as high as 90.2% after 600 cycles at 500 mA g^−1^, with a little capacity degradation. This work could provide a simple and effective way for PBA cathode materials with unique morphology, enhancing electrochemical performance.

## Data Availability

The original contributions presented in this study are included in the article/Supplementary Material. Further inquiries can be directed to the corresponding author.

## Supplementary Materials

The following supporting information can be downloaded at: https://www.mdpi.com/article/10.3390/ma18133174/s1, Figure S1. Rietveld refinements of (a) PB-1, (b) PB-2, (c) PB-3, and (d) PB-4. Figure S2. Nitrogen adsorption–desorption isotherms of (a) PB-2 and (b) PB-3. Figure S3. CV curves of different samples measured at a scan rate of 0.1 mV s−1: (a) PB-1, (b) PB-2, (c) PB-3, and (d) PB-4. Figure S4. The first two hundred cycles of dQ/dV curves of (a) PB-1, (b) PB-2, (c) PB-3, and (d) PB-4. Figure S5. Charge–discharge curves at various current densities of (a) PB-1, (b) PB-2, (c) PB-3, and (d) PB-4. Figure S6. The Nyquist plots of all samples. Figure S7. (a) SEM and (b) XRD of PB-3 before and after 400 cycles. Figure S8. Rietveld refinements of PB-3 after 400 cycles. Table S1. The element content of Na and Fe in PB-1, PB-2, PB-3, and PB-4 from ICP-MS analysis. Table S2. Structural parameters of PB-1, PB-2, PB-3, and PB-4 obtained from Rietveld analysis. Table S3. The fitting resistance values for half cells of all samples. Table S4. Comparison of the electrochemical performance of as-prepared PB-3 in this work with other reported PB-based cathodes for sodium-ion batteries. Table S5. Structural parameters of PB-3 after 400 cycles obtained from Rietveld analysis. References [19,30,37,51,52,53,54,55] are cited in the Supplementary Materials.
