# Supplementary material for: Open-Pore Skeleton Prussian Blue as a Cathode Material to Achieve High-Performance Sodium Storage
Source: Materials (Basel). 2025 Jul 4;18(13):3174. doi: 10.3390/ma18133174 (PMC12251184; doi:10.3390/ma18133174)
Supplement: Supplementary file 1 [file materials-18-03174-s001.zip › materials-3692439-supplementary.pdf]

Open-Pore Skeleton Prussian Blue as a Cathode Material to Achieve High-Performance Sodium Storage

Wenxin Song [1,†], Yaxin Li [1,†], Jiahao Chen [1], Huihua Min [2], Xinyuan Wu [1], Xiaomin Liu [1] and Hui Yang [1,*]

[1] College of Materials Science and Engineering, Nanjing Tech University, Nanjing 211816, China;
wenxinson@163.com (W.S.); liyaxin1101@163.com (Y.L.); chenjiahao1998@njtech.edu.cn (J.C.); wuxinyuan0315@163.com (X.W.); liuxm@njtech.edu.cn (X.L.)

[2] Electron Microscope Lab, Nanjing Forestry University, Nanjing 210037, China; hhmin@njfu.edu.cn

* Correspondence: yanghui@njtech.edu.cn

† These authors contributed equally to this work.

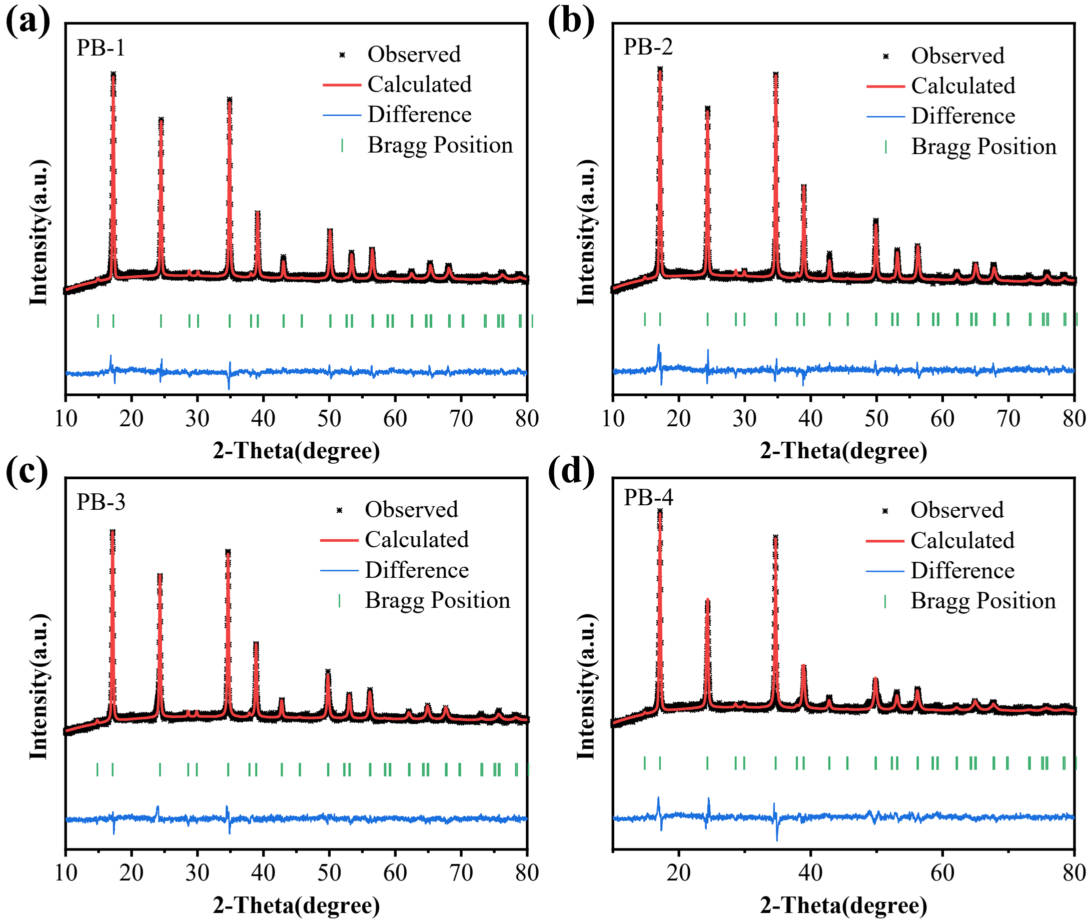

Figure S1. Rietveld refinements of (a) PB-1, (b) PB-2, (c) PB-3, and (d) PB-4.

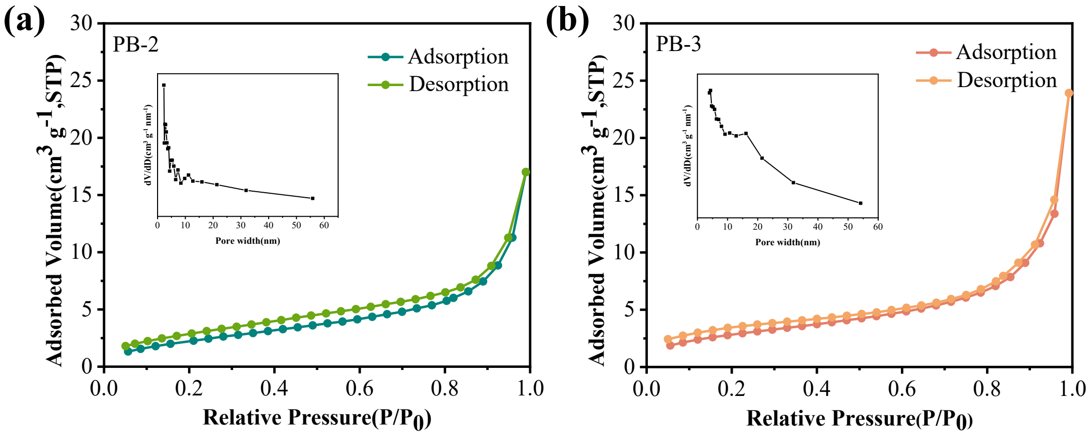

Figure S2. Nitrogen adsorption–desorption isotherms of (a) PB-2 and (b) PB-3.

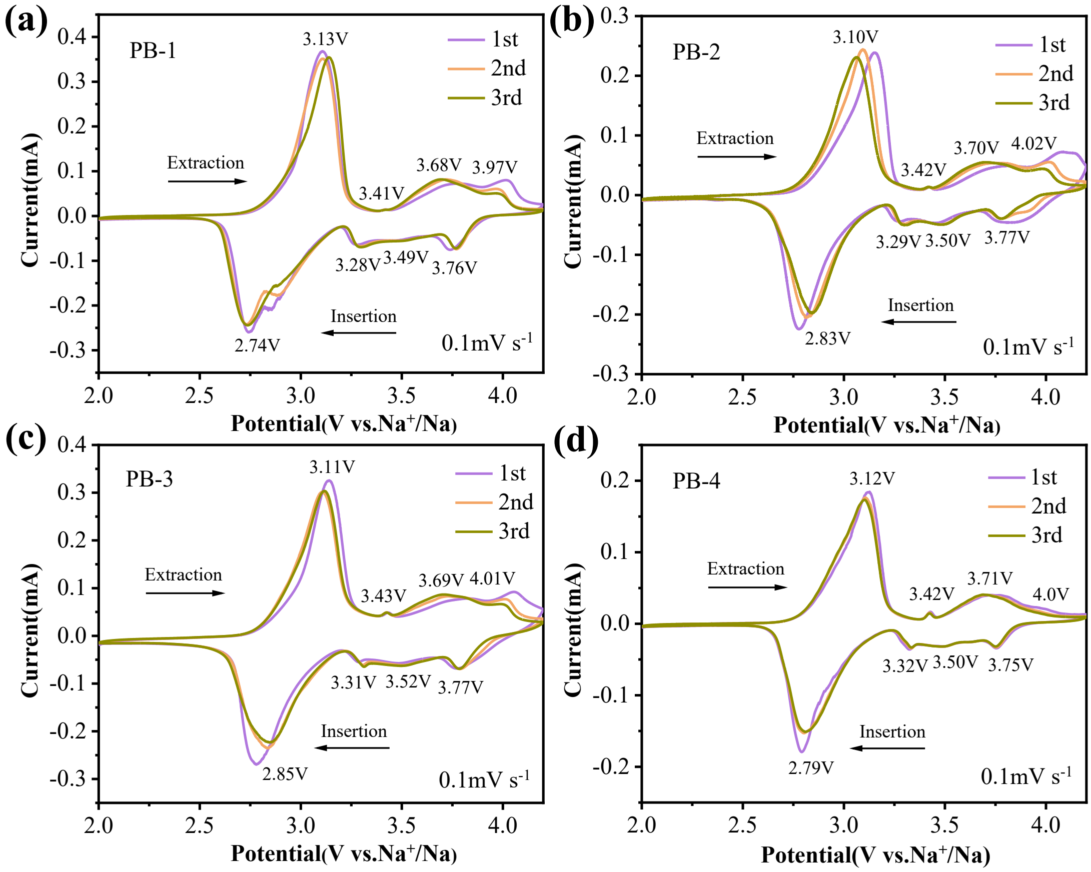

Figure S3. CV curves of different samples measured at a scan rate of 0.1 mV s−1: (a) PB-1, (b) PB-2, (c) PB-3, and (d) PB-4.

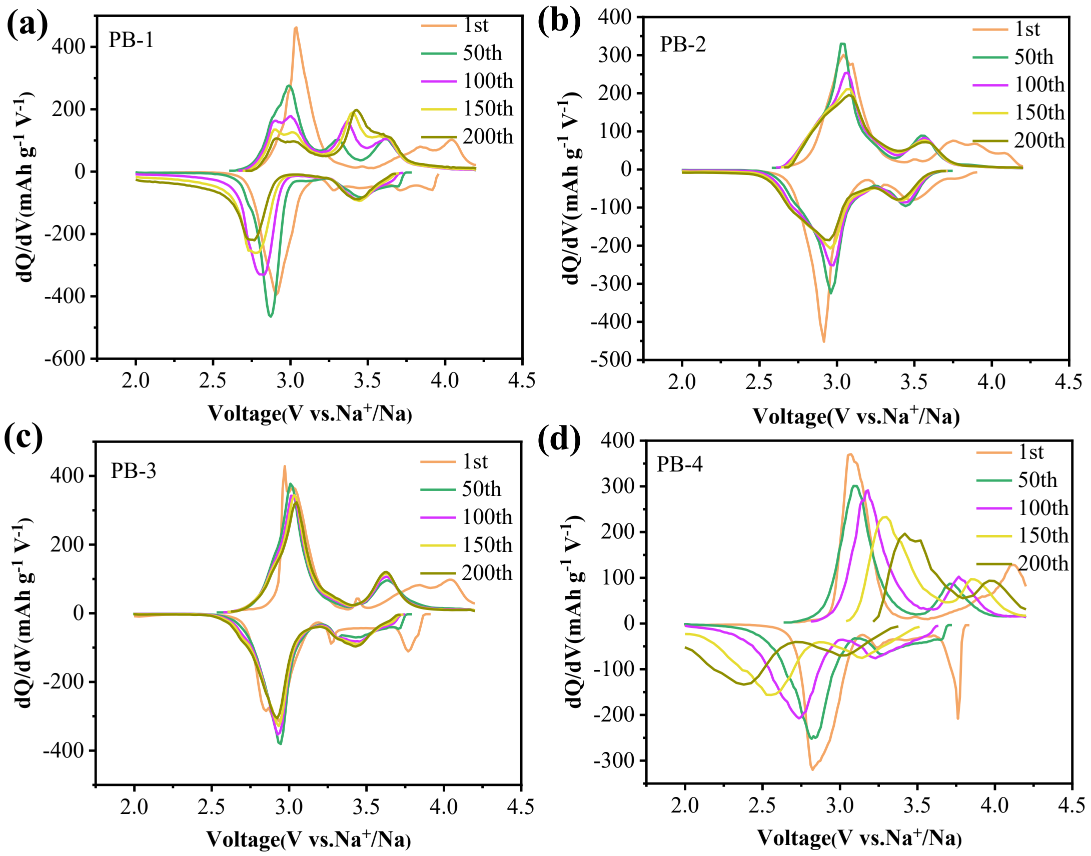

Figure S4. The first two hundred cycles of dQ/dV curves of (a) PB-1, (b) PB-2, (c) PB-3, and (d) PB-4.

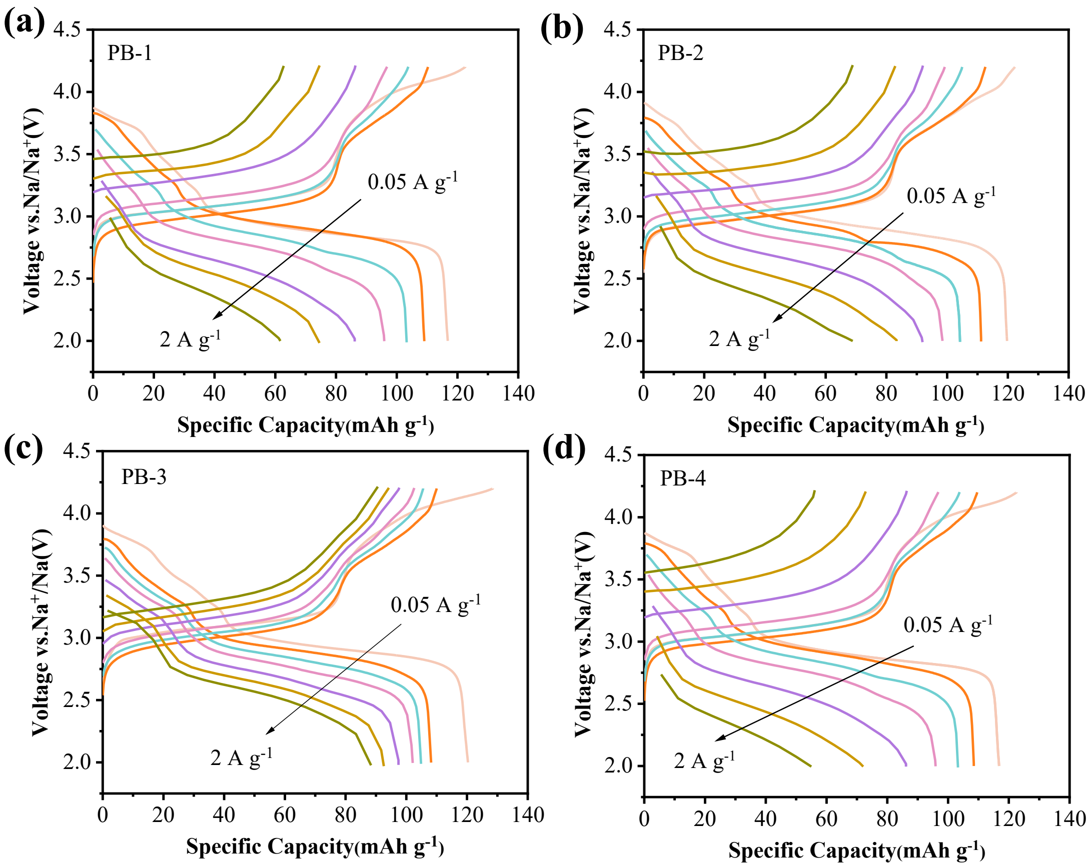

Figure S5. Charge–discharge curves at various current densities of (a) PB-1, (b) PB-2, (c) PB-3, and (d) PB-4.

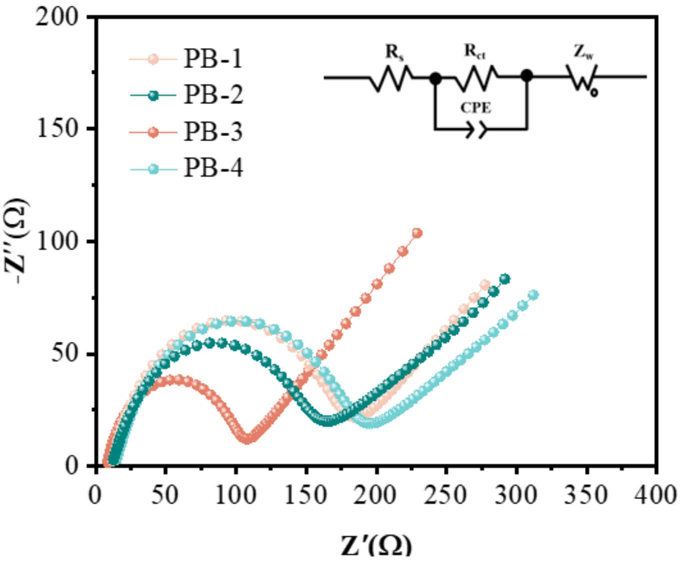

Figure S6. The Nyquist plots of all samples.

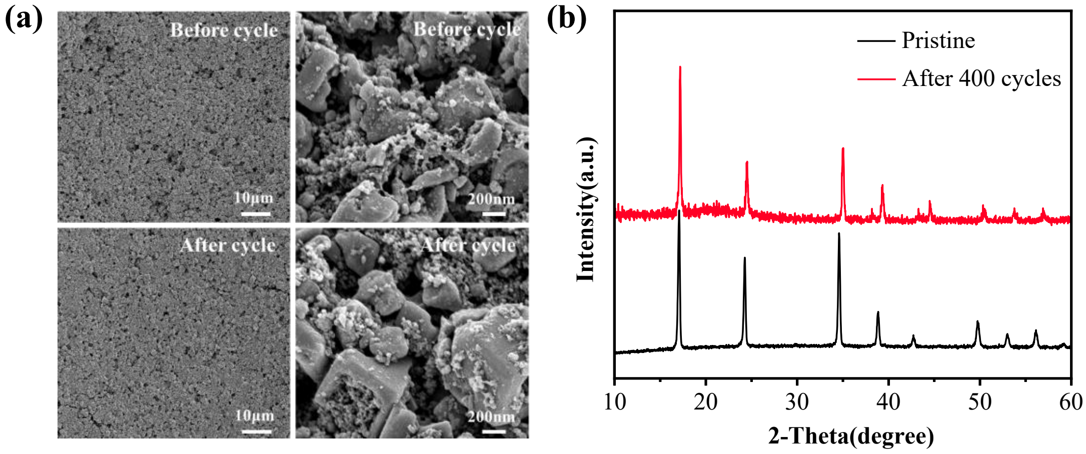

Figure S7. (a) SEM and (b) XRD of PB-3 before and after 400 cycles.

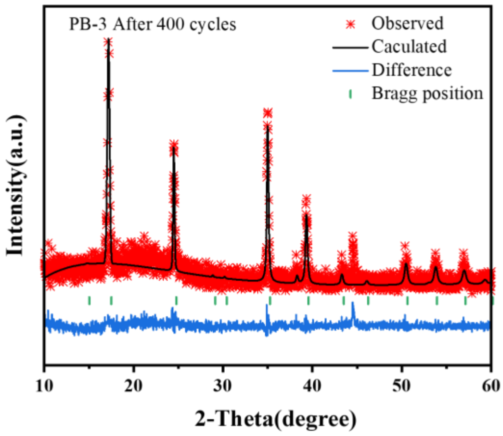

Figure S8. Rietveld refinements of PB-3 after 400 cycles.

Table S1. The element content of Na and Fe in PB-1, PB-2, PB-3, and PB-4 from ICP-MS analysis.

	Na (wt.%)	Fe (wt.%)	Na/Fe (mol)
PB-1	11.01	33.56	0.796
PB-2	11.92	33.79	0.855
PB-3	12.11	33.67	0.873
PB-4	11.73	32.92	0.864

Table S2. Structural parameters of PB-1, PB-2, PB-3, and PB-4 obtained from Rietveld analysis.

PB-1. Cubic, space group Fm-3m, a=b=c=10.2940Å, α=β=γ=90°, V= 1090.8184Å^3, R_{wp}=4.41%

Atom	x	y	z	Site	Occupation
Na	0.2500	0.2500	0.2500	8c	0.7352
C	0.1823	0.0000	0.0000	24e	0.9436
N	0.2951	0.0000	0.0000	24e	0.9436
Fe1	0.5000	0.0000	0.0000	4a	0.9436
Fe2	0.0000	0.0000	0.0000	4b	1.0000

PB-2. Cubic, space group Fm-3m, a=b=c=10.3375Å, α=β=γ=90°, V= 1104.7056Å^3, R_{wp}=4.00%

Atom	x	y	z	Site	Occupation
Na	0.2500	0.2500	0.2500	8c	0.7965
C	0.1964	0.0000	0.0000	24e	0.9582
N	0.2965	0.0000	0.0000	24e	0.9582
Fe1	0.5000	0.0000	0.0000	4a	0.9582
Fe2	0.0000	0.0000	0.0000	4b	1.0000

PB-3. Cubic, space group Fm-3m, a=b=c=10.3576Å, α=β=γ=90°, V= 1111.1620Å^3, R_{wp}=4.10%

Atom	x	y	z	Site	Occupation
Na	0.2500	0.2500	0.2500	8c	0.8215
C	0.2052	0.0000	0.0000	24e	0.9776
N	0.2983	0.0000	0.0000	24e	0.9776
Fe1	0.5000	0.0000	0.0000	4a	0.9776
Fe2	0.0000	0.0000	0.0000	4b	1.0000

PB-4. Cubic, space group Fm-3m, a=b=c=10.3225Å, α=β=γ=90°, V= 1099.9037Å^3, R_{wp}=5.84%

Atom	x	y	z	Site	Occupation
Na	0.2500	0.2500	0.2500	8c	0.7723
C	0.1985	0.0000	0.0000	24e	0.9513
N	0.2972	0.0000	0.0000	24e	0.9513
Fe1	0.5000	0.0000	0.0000	4a	0.7513
Fe2	0.0000	0.0000	0.0000	4b	1.0000

Table S3. The fitting resistance values for half cells of all samples.

Sample	PB-1	PB-2	PB-3	PB-4
R_s(ohm)	12.56	12.41	8.53	14.68
R_{ct}(ohm)	169.16	149.78	96.35	178.58

Table S4. Comparison of the electrochemical performance of as-prepared PB-3 in this work with other reported PB-based cathodes for sodium-ion batteries.

Sample	Synthesis method	Current density (mA g^{-1})	Initial capacity (mAh g^{-1})	Cycle number	Capacity Retention	Ref.
$Na_{1.64}Fe[Fe(CN)_6]_{0.98}\cdot2.67\ H_2O$	co-precipitation	500	102	600	90.2%	This work
$Na_{0.9}Fe[Fe(CN)_6]_{0.96}\cdot2.39H_2O$	ball-milling	170	120	300	79%	[1]
$Na_{1.76}Ni_{0.12}Mn_{0.88}[Fe(CN)_6]_{0.98}$	hydrothermal	100	100	800	83.3%	[2]
$Na_{1.52}FeFe(CN)_6\cdot4H_2O$	co-precipitation	117	60	150	93.2%	[3]
$Na_{1.56}Fe[Fe(CN)6]_{3.1}\cdot H_2O$	one-step	20	103	400	97%	[4]
$Na_{1.70}FeFe(CN)_6$	co-precipitation	200	120.7	100	72.4%	[5]
$Na_{1.51}Fe[Fe(CN)_6]_{0.87}\cdot1.83H_2O$	co-precipitation	17	124.2	1000	65.3%	[6]
$Na_2NiFe(CN)_6$	etching	15	83.5	1000	94%	[7]
$Na_{1.65}Co[Fe(CN)_6]_{0.88}\cdot\square_{0.12}\cdot1.1\ H_2O$	self-template	100	123.2	500	71.5	[8]

Table S5. Structural parameters of PB-3 after 400 cycles obtained from Rietveld analysis.

PB-3 after 400 cycles. Cubic, space group Fm-3m, a=b=c=10.3512Å, α=β=γ=90°, V= 1109.1036Å^3, R_{wp}=9.31%

Atom	x	y	z	Site	Occupation
Na	0.2500	0.2500	0.2500	8c	0.8212
C	0.2043	0.0000	0.0000	24e	0.9814
N	0.2993	0.0000	0.0000	24e	0.9814
Fe1	0.5000	0.0000	0.0000	4a	0.9814
Fe2	0.0000	0.0000	0.0000	4b	1.0000

Reference

[1] Y. You, X. Yu, Y. Yin, et al., Sodium iron hexacyanoferrate with high Na content as a Na-rich cathode material for Na-ion batteries, Nano Research, 8 (2015): 117-128. http://doi.org/10.1007/s12274-014-0588-7

[2] D. Yang, J. Xu, X.-Z. Liao, et al., Structure optimization of Prussian blue analogue cathode materials for advanced sodium ion batteries, Chemical Communications, 50 (2014): 13377-13380. http://doi.org/10.1039/C4CC05830E

[3] X. Wu, W. Deng, J. Qian, et al., Single-crystal FeFe(CN)6 nanoparticles: a high capacity and high rate cathode for Na-ion batteries, Journal of Materials Chemistry A, 1 (2013): 10130-10134. http://doi.org/10.1039/C3TA12036H

[4] W.-J. Li, S.-L. Chou, J.-Z. Wang, et al., Facile Method To Synthesize Na-Enriched Na1+xFeFe(CN)6 Frameworks as Cathode with Superior Electrochemical Performance for Sodium-Ion Batteries, Chemistry of Materials, 27 (2015): 1997-2003. http://doi.org/10.1021/cm504091z

[5] Y. Liu, Y. Qiao, W. Zhang, et al., Sodium storage in Na-rich NaxFeFe(CN)6 nanocubes, Nano Energy, 12 (2015): 386-393. http://doi.org/10.1016/j.nanoen.2015.01.012

[6] C.-M. Xu, J. Peng, X.-H. Liu, et al., Na1.51Fe[Fe(CN)6]0.87·1.83H2O Hollow Nanospheres via Non-Aqueous Ball-Milling Route to Achieve High Initial Coulombic Efficiency and High Rate Capability in Sodium-Ion Batteries, Small Methods, 6 (2022): 2200404. http://doi.org/10.1002/smtd.202200404

[7] Y. Zhao, J. Peng, K. Chen, et al., Boosting the sodium storage performance of Prussian blue analogues via effective etching, Science China Chemistry, 66 (2023): 3154-3160. http://doi.org/10.1007/s11426-023-1824-3

[8] T. Huang, Y. Niu, Q. Yang, et al., Self-Template Synthesis of Prussian Blue Analogue Hollow Polyhedrons as Superior Sodium Storage Cathodes, ACS Applied Materials & Interfaces, 13 (2021): 37187-37193. http://doi.org/10.1021/acsami.1c09678
